# Comprehensive genetic testing for primary immunodeficiency disorders in a tertiary hospital: 10-year experience in Auckland, New Zealand

**DOI:** 10.1186/s13223-016-0169-2

**Published:** 2016-12-07

**Authors:** See-Tarn Woon, Rohan Ameratunga

**Affiliations:** Department of Virology and Immunology, LabPLUS, Auckland City Hospital, Grafton, Auckland, 1148 New Zealand

**Keywords:** Primary immunodeficiencies, Genetic testing, Next generation sequencing

## Abstract

**Background and purpose:**

New Zealand is a developed geographically isolated country in the South Pacific with a population of 4.4 million. Genetic diagnosis is the standard of care for most patients with primary immunodeficiency disorders (PIDs).

**Methods:**

Since 2005, we have offered a comprehensive genetic testing service for PIDs and other immune-related disorders with a published sequence. Here we present results for this program, over the first decade, between 2005 and 2014.

**Results:**

We undertook testing in 228 index cases and 32 carriers during this time. The three most common test requests were for X-linked lymphoproliferative (XLP), tumour necrosis factor receptor associated periodic syndrome (TRAPS) and haemophagocytic lymphohistiocytosis (HLH). Of the 32 suspected XLP cases, positive diagnoses were established in only 2 patients. In contrast, genetic defects in 8 of 11 patients with suspected X-linked agammaglobulinemia (XLA) were confirmed. Most XLA patients were initially identified from absence of B cells. Overall, positive diagnoses were made in about 23% of all tests requested. The diagnostic rate was lowest for several conditions with locus heterogeneity.

**Conclusions:**

Thorough clinical characterisation of patients can assist in prioritising which genes should be tested. The clinician-driven customised comprehensive genetic service has worked effectively for New Zealand. Next generation sequencing will play an increasing role in disorders with locus heterogeneity.

## Background

Failure to effectively combat infections is a hallmark of immunodeficiencies (IDs) such as primary immunodeficiency disorders (PIDs) where rare genetic defects lead to compromised host defences. Affected patients are prone to recurrent and severe infections [1]. Some patients develop autoimmunity and malignancy because of immune dysregulation [2, 3]. The severity of these disorders ranges from asymptomatic IgA deficiency to life threatening conditions such as severe combined immunodeficiency (SCID). The majority of PIDs are monogenic disorders. Almost 300 genetic defects have so far been identified [4]. A delayed diagnosis can impact on prognosis if a patient has a life threatening disorder, such as presymptomatic males with X-linked lymphoproliferative syndrome (XLP) from SH2D1A or BIRC4 mutations [5].

New Zealand is a developed geographically isolated country with a population of 4.4 million in the South Pacific. A dedicated comprehensive customised genetic testing service for PIDs was established in 2005 [6]. The primary aim of the service was to offer rapid genetic testing for any PID disorder with a published sequence. The service initially offered genetic testing for XLP and X-linked agammaglobulinemia (XLA). Over time, the scope of the service has broadened to include patients with other PIDs as well as haemophagocytic lymphohistiocytosis (HLH) and atypical haemolytic uremic syndrome (aHUS) and periodic fever/autoinflammatory syndromes.

HLH is a rare but potentially fatal disease of uncontrolled immune activation. Genetic testing for HLH overlaps with testing for XLP. Similarly, genetic testing plays an important role in patients with aHUS. Identification of the specific genetic defect impacts on clinical management of aHUS patients. Membrane cofactor protein (MCP, CD46) is a complement protein that protects organs from injury by complement. Mutations in MCP are associated with aHUS [7]. Patients with terminal renal failure can undergo successful renal transplantation if they only have an MCP mutation [8]. The transplanted kidney expresses normal levels of MCP and is protected against complement mediated damage. In contrast mutations of factor H and I could damage a transplanted kidney, hence the importance of undertaking genetic studies in patients with aHUS. This is particularly important given that eculizumab is not funded in New Zealand.

The service provides testing for periodic fever syndromes. This group of disorders include cryopyrin-associated periodic syndrome (CAPS), tumor necrosis factor receptor associated periodic syndrome (TRAPS), familial Mediterranean fever (FMF) and mevalonate kinase deficiency (MKD). Identification of the exact genetic defect in these autoinflammatory disorders is very important as the treatments vary.

Tertiary hospitals offering PID testing services in developed and developing countries have reported a wide spectrum of PIDs within their respective communities [9–12]. Access to specialised clinical and laboratory resources differ among the countries, depending on the expertise and financial resources of each healthcare system [13]. We have made the case for a national clinical service for PIDs in New Zealand [14]. It is hoped the customised genetic testing service will be integrated into a future national PID service.

In this report, we review the results of patients referred to the LabPlus comprehensive customised genetic testing service between 2005 and 2014. We also discuss the implications of future genetic testing with the advent of next generation sequencing (NGS).

## Methods

The customised genetic testing service is clinician-driven and a test is rapidly developed if there is an appropriate clinical request. The service typically offers results within a week for smaller genes and up to three weeks for longer genes. In addition to the rapid turnaround time, it has also reduced the need to send samples to overseas laboratories. For most of the genes tested, it has been much cheaper to undertake mutation analysis in New Zealand, compared with overseas laboratories. Twice weekly meetings are held to discuss cases and to review the results of other tests including protein-based assays and flow cytometry. The genetic testing strategy is based on detailed clinical and phenotypic data.

The service follows the guidelines for molecular diagnostic laboratories issued by the Centres for Disease Control [15] and is accredited by IANZ (International Accreditation New Zealand). The service has a yearly DNA sample exchange with overseas laboratories as part of the external quality assurance program. We are not aware of a formal external quality assurance program for PID genetic testing by organisations such as the College of American Pathologists.

Patients receive genetic counselling before blood is drawn. Results of testing are only meaningful if interpreted in the appropriate clinical context. As we show here, close consultation with clinicians increases the success rate of testing as it helps prioritise genes for testing, based on phenotype. We have thus maintained a close working relationship with requesting clinicians, which has obviated the need for testing algorithms.

Reference gene sequences are downloaded from public databases such as Genatlas and Ensembl. Intronic primers flanking exon regions are designed using Oligo version 6.44 (Molecular Biology Insights, Cascade, CO, USA) or Primer3 (SimGene.com). Primer sequences are also obtained from published literature. M13 adapter sequences are added to the 5′ terminus of primer sequences before submission for synthesis.

Genomic DNA is prepared from whole blood with the Puregene DNA purification kit (Gentra Systems, Minneapolis, MN, USA) [16]. PCR of genomic DNA is performed using primers and cycling conditions as described in Roche FastStart Taq DNA polymerase instruction manual. Amplicons are treated with Illustra ExoProStar (GE Healthcare Life Sciences, Little Chalfont, UK). The sequencing reactions are performed with 5 pmol primers and BigDye^®^ terminator cycle sequencing (Applied Biosystems, Foster City, CA). Sequencing products are added to Agentcourt CleanSeq (Beckman Coulter, Brea, CA), washed twice in 85% ethanol and analysed on an ABI PRISM^®^ 3130xl Genetic Analyzer.

Genetic variants (single nucleotide polymorphisms and small insertions or deletions) are identified using SeqMan 5.01 (DNASTAR, Madison, WI, USA) and referenced to respective allele frequencies determined in large population studies (1000 Genomes and HapMap). Variants present in less than 1% of the healthy population are further analysed. We evaluate the in silico effect of amino acid substitution in non-synonymous SNPs using prediction software Polyphen2 and SIFT. Clinical variants are also verified in ClinVar, disease specific registries/databases and literature review.

Testing for F12 mutations (c.1032C > A, Thr328Lys) for patients with HAE with normal C1 inhibitor levels (HAE type III) was undertaken by Sonic Laboratories in Sydney.

## Results

**Fig. 1:**
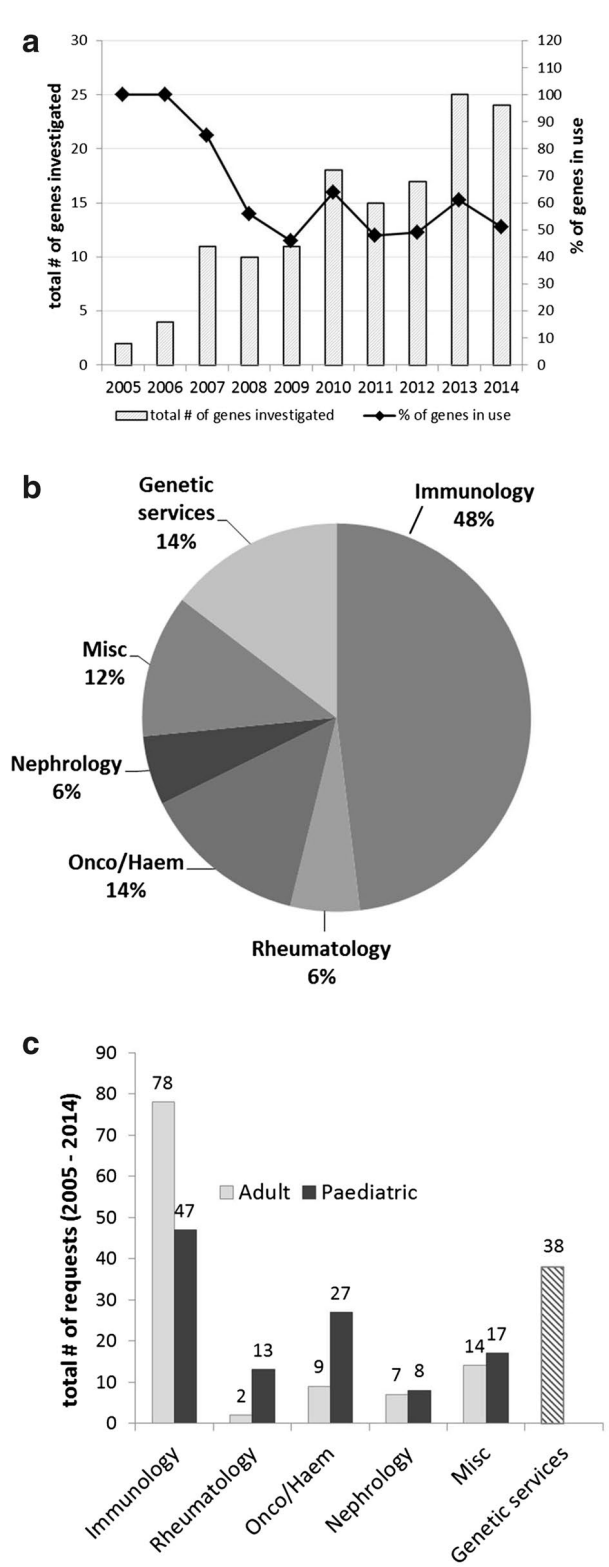
**a** Number of gene tests requested annually (2005–2014), **b** Breakdown of different hospital services requesting genetic tests (2005–2014), **c** Paediatric and adult services of different specialities requesting genetic testing (2005–2014)

The service has experienced a steady increase in the number of gene testing requests in the 10-year period (Fig. 1a). At least 3 new genes were added to the test guide every year since 2007. We undertook at least half of the available gene tests on offer annually. As of 2014, the service offered gene tests for 30 PID genetic disorders.

We carried out testing on 228 index cases and 32 carriers in 2005–2014. Test requests were received from various clinical services in Auckland City Hospital and other hospitals around New Zealand (Fig. 1b). Almost half of the referrals came from the two public hospital immunology services in Auckland. The adult immunology service at Auckland City Hospital referred most of the tests and the remaining were from the paediatric immunology (Fig. 1c). Referrals from genetic services were usually for family studies and patients requiring carrier status testing. The types of requests from the paediatric oncology/haematology service were diverse. Approximately 40% of the requests were for suspected lymphoproliferative disorders such as XLP or HLH.

The most frequent requests from the adult immunology service were for TRAPS or hereditary angioedema (HAE). HAE patients were either tested for mutations of the C1 inhibitor (C1-INH, SERPING1) gene and/or F12. We did not identify any F12 mutations in patients with HAE with normal C1 inhibitor levels. We now only recommend testing if the angioedema does not respond to high dose antihistamines and the patient has normal complement studies.

From the paediatric immunology service, there were several requests for chronic granulomatous disease (CGD) testing. The causative genes in X-linked and autosomal recessive CGD were also tested in siblings and parents. SCID gene testing was requested by both paediatric immunology and oncology/haematology services. These two services also requested testing for patients with suspected lymphoproliferative disorders including XLP and HLH. As expected, most requests for aHUS were from the paediatric nephrology service.

Fifty three index patients (23% of the 228 patients) had positive genetic diagnoses (Table 1). A strong correlation between phenotype and genotype exists for certain conditions. The rate of positive genetic diagnoses varied for different disorders. A high rate of genetic diagnosis was confirmed in patients with XLA, autosomal recessive CGD, ectodermal dysplasia and immunodeficiency (EDA-ID), Griscelli type 2 and Shwachman–Diamond syndrome (SDS).

**Table 1:** Genetic testing results of patients referred to molecular immunology service (2005–2014)

Test	Genes	Patients	Carriers?	Index patients tested positive^b^	% tested positive
aHUS/C3 glomerulopathy	CD46	15		1	7
	CFH			1	7
	CFI			0	0
ALPS	CD95	7		0	0
APECED	AIRE	1		0	0
CGD-AR	NCF1	8	4	4	50
CGD-XL	CYBB	3	1	3	100
CHARGE	CHD7	1		0	0
C2 deficiency	C2	1		0	0
CAPS	NLRP3/CIAS1	9	2	3	33
DOCK8 deficiency	DOCK8	1		0	0
EDA-ID	NEMO	1		1	100
Griscelli type 2	RAB27A	3		3	100
HAE	SERPING1	13	3	6	46
HAE type III^a^		17		0	0
HLH	Perforin	20		1	5
	UNC13D			1	5
	STXBP2			0	0
	STX11			0	0
HIM-XL	CD40L	12	2	3	25
HIM	AICDA	4		0	0
	UNG			0	
	AUG			0	
	CD40			0	
Hyper IgE	STAT3	9		4	44
IPEX	FoxP3	1		0	0
LPD-AR (EBV driven)	ITK	1		0	0
LPD-XL (XLP)	SH2D1A	32	9	1	3
	BIRC4			1	3
Netherton syndrome	SPINK5	1		0	0
Periodic fever syndrome	MEFV	11		2	18
	MVK			0	0
Properdin deficiency	properdin	1		0	0
SCID-AR	JAK3	2	2	1	11
	RAG1 & 2	3		0	0
	ADA	0		0	0
	LIG4	1		0	0
	Artemis	1		0	0
	Cern. Factor^c^	1		0	0
	IL-7R	1		0	0
SCID-XL	IL2-RG	4	1	2	50
SDS	SBDS	1	2	1	100
TRAPS	TNFRSF1A	19		2	11
UNC93b deficiency	UNC93b	1		0	0
WAS	WASP	10		5	50
WHIM syndrome	CXCR4	1		0	0
XLA	BTK	11	4	8	73
Total		228	30	53	23

*aHUS* atypical haemolytic uremic syndrome; *ALPS* autoimmune lymphoproliferative syndrome; *APECED* autoimmune polyendocrinopathy type 1; *CGD*-*XL* X-linked chronic granulomatous disease; *CGD*-*AR* autosomal recessive chronic granulomatous disease; *CHARGE* coloboma, heart defect, atresia choanae, retarded growth and development, genital abnormality, and ear abnormality; *CAPS* cryopyrin-associated periodic syndrome; *EDA*-*ID* ectodermal dysplasia and immunodeficiency; *HAE* hereditary angioedema; *HAE type III* type 3 hereditary angioedema; *HLH* hemophagocytic lymphohistiocytosis; *HIM*-*XL* hyper immunoglobulin M syndrome, X-linked; *HIM* hyper immunoglobulin M syndrome; *IPEX* immune dysregulation, polyendocrinopathy, enteropathy, X-linked syndrome; *LPD*-*AR* lymphoproliferative disorder, autosomal recessive; *LPD*-*XL* lymphoproliferative disorder, X-linked; *SCID*-*AR* autosomal recessive severe combined immune deficiency; *SCID*-*XL* X-linked severe combined immune deficiency; *SDS* Shwachman-Diamond syndrome; *TRAPS* TNF receptor-associated periodic syndrome; *WAS* Wiskott-Aldrich syndrome; *WHIM* warts, hypogammaglobulinemia, infections, and myelokathexis; *XLP* X-linked lymphoproliferative syndrome; *XLA* X-linked agammaglobulinemia

^a^DNA from patients with suspected factor XII mutation were sent to Sonic laboratories in Sydney

^b^Mutations of genes tested positive for disorders were described in Table 2

^c^Cernunnos factor

Eight of the 11 patients with suspected XLA were positively identified by mutational analysis (Table 1). XLA was initially suspected by the almost complete absence of peripheral blood B cells. XLA patients were tested to establish the exact mutation in the Bruton’s tyrosine kinase (BTK) gene to complete their laboratory profiles. The genetic results did not immediately affect patient management since these patients were already on intravenous or subcutaneous immunoglobulin therapy. Identification of the mutation has profound implications for carrier female family members considering preimplantation genetic diagnosis or chorion villus sampling.

The rates of genetic diagnoses were lowest for some conditions with locus heterogeneity. The genotype-phenotype correlation is poor for patients with suspected HLH, aHUS or periodic fever syndromes (Table 1). Furthermore, some disorders may have a phenocopy such as patients with anti-Factor H antibodies leading to aHUS [17]. This can contribute to a lower frequency of genetic diagnosis in some disorders.

Protein-based assays can be useful for investigating patients without confirmed genetic diagnoses in other conditions. Our cohort of 7 patients suspected of ALPS did not have detectable germline FAS mutations. cDNA analyses indicated productive FAS gene expression. Two patients are likely to have ALPS based on clinical presentation, presence of elevated double negative alpha beta positive T cells and defective apoptotic cell function. Assessment of germline FasL gene and FAS gene on sorted double negative T cells in patients without FAS mutations could further strengthen our testing strategy in the future.

## Discussion

We have tabulated our patient results according to the suspected genetic diagnosis and the mutations identified (Tables 1, 2). The PID cases we encountered were varied akin to those elsewhere, albeit at a lower number due to New Zealand’s small population size.

**Table 2:** Mutations detected in patients referred to the service (2005–2014)

Test	Genes	Mutation	References
aHUS/C3 glomerulopathy	MCP, CFH, CFI	CFH: c.3120delT, F960Xfs MCP: c.995_996delAG, S274fsX284	[7, 29]
CGD-XL	NCF1	c.87_88delGT, c.271G > A (V25FsX51, R90H) (2 patients) no NCF1 gene, only pseudogene present (2 patients)	[30, 31]
CGD-AR	CYBB	c.1461 + 1G > A, IVS11 + 1G > A deletion of exons 9–13 c.987C > A, C329X	[32, 33]
CAPS	NLRP3/CIAS1	c.913G > A, D305 N c.2113C > A, Q705 K c.920T > C, L307P	[34, 35]
EDA-ID	NEMO	c.742G > C, A162P	[36]
Griscelli type 2	RAB27A	c.550C > T, R184X	[37]
HAE	C1-INH/serping1	c.188C > T, S63F c.539A > C, Q180P c.1342G > T, E488X c.1396C > T, R466C c.1089delG, K364fsX32 c.1034_1035insCCAC, Q346fsX369	[38–41]
HLH	perforin, UNC13D	perforin: c.272C > T (A91 V), UNC13D: c.175G > A (A59T)	[42]
HIM-XL	CD40L	c.475G > A, W140X c.147delG, R49fsX53	[43]
Hyper-IgE	STAT3	c.2115C > T, Q633X c.1909G > A, V637 M c.1235C > A, T412 N c.2134G > C, V712L	[44, 45]
Periodic fever syndrome	MEFV, MVK	MEFV: c.442G > C, c.1105C > T (E148Q, P369S) MEFV: c.1105C > T, c.1223G > A (P369S, R408Q)	[46, 47]
SCID-AR	JAK3	c.1351C > T, c.2148G > A (R451X, W716X)	[48]
SCID-XL	IL2-RG	c.677G > A, R226H c.196A > C, Q61P	[49]
SDS	SDSP	c.184A > T (K62X), 258 + 2T > C (IVS2 + 2T > C)	[50, 51]
TRAPS	TNFRSF1A	c.362G > A, R121Q (low penetrance SNP, 2 patients)	[52]
WAS	WASP	c.431G > A, E133 K (2 patients) c.257G > A, R86H c.523_524delAG, R156fsX167 c.134C > T, R38X	[53–55]
XLP	SH2D1A, BIRC4	SH2D1A: c.261delT, Q88fsX95 BIRC4: c.598_600delTGC, C200del	[56, 57]
XLA	BTK	c.1691G > A, R520Q (2 patients) c.1100C > A, A367E c.1906_1908GAG > TTT, E636F c.1581_1584delTTTG, C527fsX528 (2 patients) 1567-2A > C (IVS15-2A > C) 776 + 1G > A (IVS8 + 1G > A)	[58, 59]

Our data supports a flexible diagnostic strategy, based on the clinical presentation. Sanger sequencing can be undertaken in cases with a well characterised phenotype such as XLA. In contrast, the genetic causes have been poorly characterised in HLH [18]. This makes genetic testing difficult and is a strong argument for NGS with gene panels, as well as screening with functional studies.

Our study has shown there are however some examples of locus heterogeneity where Sanger sequencing can be useful. Five causative genetic defects have been identified in patients with CGD (Table 2). Clinical data including family history and relative severity may offer clues to the nature of the affected gene. An X-linked inheritance pattern might suggest CYBB gene mutations, while a relatively mild phenotype might suggest a mutation in the NCF1 gene. This illustrates the value of close collaboration between requesting clinicians and the laboratory.

Our experience has shown that variants of unknown clinical significance (VUS) can be a major problem. Patients should undergo genetic counselling before testing so their expectations can be managed. Testing may not identify the causative gene in all patients and the presence of VUS can place both patient and referring physician in a difficult position. As described below, VUS will become a much more significant problem with the advent of NGS.

It is clear there will be major changes in genetic testing strategies in the near future. The approach to genetic testing described here can be time consuming and expensive. Traditionally clinicians undertake serial testing of candidate genes or order a gene panel. As shown here the diagnostic rates are lower for most conditions for which there is locus heterogeneity. NGS offers a possible solution for such conditions.

We have gained experience with WES in the last five years. Patients with undiagnosed B cell deficiencies were offered the opportunity to participate in our common variable immunodeficiency disorder (CVID) research program. Patients were accepted for the study if they fulfilled the Ameratunga et al. criteria for CVID [14, 19, 20]. Written consent was sought prior to enrolment in the project.

Two kindreds enrolled in the CVID program underwent WES trio analyses resulting in confirmed diagnoses [21]. These genetic defects would not have been discovered by targeted NGS since these genes were previously not described in PID patients. It is likely many CVID cases will not have a genetic diagnosis following targeted NGS [22]. We have not offered routine diagnostic testing for CVID partly due to its locus heterogeneity and variable clinical phenotypes due to unknown environmental and genetic factors. Furthermore, the significance of sequence variations in genes encoding molecules such as TACI and BAFFR are uncertain [20, 23].

If a patient presents with a disorder such as HLH, there is a reasonable argument for undertaking WES as the first test. If a defective gene is not found, patients could be referred for gene discovery using WES and/or whole genome sequencing (WGS) with appropriate consent after ethics approval. We are thus adapting our testing strategy to the phenotype of the patient and the family.

Risk alleles add a further level of complexity to diagnostic testing for aHUS. Several studies have shown that sequence variants of complement-associated proteins can predispose to aHUS [17]. The precise risk is uncertain but significantly complicates decisions regarding renal transplantation. These alleles could be analysed as part of a WES panel. Unfortunately, many countries including New Zealand cannot afford the extremely high cost of eculizumab, which is very effective in treating and preventing aHUS exacerbations. It is hoped that cheaper biosimilars will become available in the near future. This may reduce the critical importance of genetic testing for aHUS.

NGS however has significant limitations, which must be appreciated before introduction into a routine diagnostic laboratory. At this time WES cannot identify large deletions or complex mutations. Coverage issues can lead to either false positive or false negative results. WGS overcomes many of these technical limitations but is not widely available for diagnostic purposes at this time. A WGS CVID study highlighted the complexity of CVID as a polygenic disorder [24]. Considerable expertise and time is required for WGS bioinformatic analysis and is presently beyond the capability of a routine diagnostic laboratory. Patients could enrol in a WGS research program if Sanger and targeted NGS failed to yield conclusive results. In these cases, the diagnosis rate is 8% (K. Gilmour, personal communication, October 14, 2016).

Targeted gene sequencing could potentially serve as an interim solution for diagnostic purposes. Targeted NGS or gene panels are less complicated and laborious than WES or WGS. The diagnostic rate for unsolved cases using gene panels have been reported in the range of 15–25% [22, 25–27]. Most of these cases were atypical clinical presentations of known PIDs. The main disadvantage of this strategy is the requirement to update gene panels and to modify the target gene enrichment process when new PID genes are listed in the latest IUIS PID classification.

In spite of its limitations, WES provides a cost effective solution to rapidly sequence large numbers of genes. Maffucci et al. [28] demonstrated the utility of WES and targeted gene filtering in achieving 30% success rate in a cohort of 50 well characterised CVID patients.

A clinical exome approach with a flexible PID gene filtering and analysis strategy is better suited for a small country such as New Zealand since updating targeted NGS gene panels can be expensive and time consuming. The gene filters allow streamlined searches for disease-causing variants and could be periodically updated for in silico investigation as new gene discoveries are made. Our laboratory is in the process of introducing gene panels for various phenotypes. Gene panels are being created for SCID, HLH, aHUS and other disorders with locus heterogeneity. It is hoped turnaround time can be shortened once these tests are undertaken in New Zealand.

It is also important to periodically review patients for whom a genetic diagnosis has not been established. As seen in Table 1 there were many requests for XLP in patients with severe EBV infections. Some of these patients may have one of the recently described “EBVopathies” such as mutations of CTLA4. Such patients without a genetic diagnosis after initial Sanger sequencing, could be offered NGS for gene discovery after the appropriate consent.

## Conclusions

We conclude the customised diagnostic PID genetic testing service has allowed us to rapidly undertake genetic testing when requested. Genetic testing will become more complex with NGS, as multiple sequence variants will be identified. Continued close involvement of referring clinicians in the testing process will be paramount. Regular meetings with requesting clinicians and analysis of other protein-based data will be crucial in determining the significance of these genetic variants.

## Abbreviations

aHUS: atypical haemolytic uremic syndrome
CAPS: cryopyrin-associated periodic syndrome
CGD: chronic granulomatous disease
EDA-ID: ectodermal dysplasia and immunodeficiency
FMF: familial mediterranean fever
HAE: hereditary angioedema
HLH: haemophagocytic lymphohistiocytosis
IANZ: International Accreditation New Zealand
IDs: immunodeficiencies
MCP: membrane cofactor protein
MKD: mevalonate kinase deficiency
NGS: next generation sequencing
PID: primary immunodeficiency disorder
SCID: severe combined immunodeficiency
SDS: Shwachman–Diamond syndrome
SNP: single nucleotide polymorphism
TRAPS: tumour necrosis factor receptor associated periodic syndrome
VUS: variants of unknown clinical significance
WGS: whole genome sequencing
XLA: X-linked agammaglobulinemia
XLP: X-linked lymphoproliferative syndrome
